# Tubercles of the Serous Membranes

**Published:** 1843-05-27

**Authors:** 


### TUBERCLES OF THE SEROUS MEMBRANES.

In a communication published in the "Archives Générales de Médecine," M. Briquet, the physician to the Hôpital Cochin, narrates three cases of tubercles and encephaloid tumors on the serous membranes terminating fatally, which occurred in his practice in the hospital, and from which he draws certain deductions.

Bayle was the first who described the formation of these heterologous productions in a precise manner. He mentions the hard and rounded granulations which are formed on the peritoneum as a consequence of chronic peritonitis, and appear to constitute a part of the serous membrane, but can be removed by scraping with a scalpel. He considered them to be merely a transformation of the exuded matter. Broussais and Laennec have also described such cases, as well as Cruveilhier, Andral, Louis, Lombard, and Chomel. As far, then, as pathological anatomy is concerned, these granulations are well known, but much uncertainty and difference of opinion exists among medical men with reference to their pathology, more especially as regards the causes that produce them. By some they are looked upon as a direct effect of inflammation, while by others they are supposed to spring from the false membranes secondarily, and by a process which has not any affinity with inflammation. It has also been believed that these heterologous productions are found in the serous membranes only after the system has been affected for a long time, and evidently modified by the existence of a similar disease in one of the principal organs. On both points M. Briquet expresses a decided opinion, he regarding the tubercular and encephaloid formation on the serous membranes as the direct result of inflammation, and fully capable of appearing previous to a similar condition obtaining in any part of the organic system. He supports these opinions by the detail of cases which he has treated, and by others selected from the works of Broussais and Andral. That they are the results of inflammation, and that of a chronic character, he concludes, because they are always situate on the free surface of the serous membranes, that they are found almost exclusively on those membranes, which have been the seat of pain and other symptoms of phlogosis, and in the greatest number in that part of the membrane which showed the strongest signs of inflammatory action, and finally, that they do not exist in the false membranes, but spring directly from the surface of the serous membrane, there not being any false membrane in or about them by which they could be enveloped, or of which they might have been a transformation.

Inflammation of the serous membranes, inducing the formation of heterologous productions, is commonly accompanied by dropsy, presenting symptoms and progress so characteristic as to afford a means of diagnosis. The first class of symptoms which present themselves have reference to the phlegmasia, the others to the progress of the effusion. If the disease commence in the chest, there is experienced a more or less severe pain in the side during repose, and on drawing a deep breath; percussion and auscultation next indicate the existence of a fluid in the cavity of the pleura, which is shown by the bronchial respiration, and the modification of the voice, to have been formed very rapidly. If the abdomen be first affected, tension of the parietes is observed, with more or less fixed pain, and constant sensibility on pressure. Dulness on percussion is also experienced in one part of the abdomen, and which continues the same, however the position of the patient be changed. Finally, the tumefaction of the abdomen is irregular, and fluctuation is not perceived until long after the commencement of the disease. The phenomena indicative of the dropsical effusion are equally characteristic, the chief being the long-continued existence of a collection of fluid in the serous cavities, before the occurrence of general anasarca. This state not being in accordance with the usual progress of dropsies, Dr. Briquet thinks it may be regarded as characteristic of the effusion following tubercular or encephaloid disease.

With respect to treatment, M. Briquet is inclined to rely almost entirely on the repeated application of blisters. Tonics and stimulants he has found aggravate the disease, while, although its cause be of an inflammatory nature, the complaint is so modified by the heterologous formations as to admit of the antiphlogistic plan being adopted only as a palliative.—*Provincial Medical Journal.* March 11, 1843.

